# Reliability of Margin Assessment after Surgery for Extremity Soft Tissue Sarcoma: The SSG Experience

**DOI:** 10.1155/2012/290698

**Published:** 2012-06-18

**Authors:** Clement S. Trovik, Sigmund Skjeldal, Henrik Bauer, Anders Rydholm, Nina Jebsen

**Affiliations:** ^1^Musculoskeletal Tumour Center, Department of Orthopedics/Oncology, Haukeland University Hospital, 5021 Bergen, Norway; ^2^Department of Orthopedics, Oslo University Hospital, Radiumhospitalet, 0310 Oslo, Norway; ^3^Division of Orthopedics, Department of Molecular Medicine and Surgery, Karolinska Institute, Karolinska University Hospital, 17176 Stockholm, Sweden; ^4^Department of Orthopedics, Institute of Clinical Sciences, Skane University Hospital, Lund University, 22185 Lund, Sweden

## Abstract

Surgery remains the mainstay of soft tissue sarcoma (STS) treatment and has been the primary treatment for the majority of patients in Scandinavia during the last 30 years although the use of adjuvant radiotherapy has increased. Patient and treatment characteristics have been recorded in the Scandinavian Sarcoma Group (SSG) Register since 1987. When the effect of new radiotherapy guidelines from 1998 was evaluated, the reliability of surgical margin assessments among different Scandinavian institutions was investigated. Margins were reevaluated by a panel of sarcoma surgeons, studying pathology and surgical reports from 117 patients, randomly selected among 470 recorded patients treated between 1998–2003. In 80% of cases, the panel agreed with the original classification. Disagreement was most frequent when addressing the distinction between marginal and wide margins. Considered the element of judgment inherent in all margin assessment, we find this reliability acceptable for using the Register for studies of local control of STS.

## 1. Introduction

Soft Tissue Sarcomas (STSs) are optimally removed with a safety margin of healthy tissue encompassing the tumor. After surgery, the completeness of removal is evaluated by assessing the quality and thickness of this margin. During the last decades, the margin has most often been classified as intralesional, marginal, wide, or radical/compartmental referring to Enneking et al. [1].

During the early years (1970s) of the Scandinavian Sarcoma Group's (SSG) existence, compartmental excisions according to Enneking were sometimes attempted. However, better referral practices, with more patients referred to tumor centers before surgery, has often made it possible to avoid the sacrifice of function such operations entail. Routine use of MRI in planning has enabled safe resection margins inside compartments [2–4]. The surgical goal is currently a wide margin with a cuff of healthy tissue surrounding the tumor. For strictly intramuscular tumors, this margin is often obtained by myectomy [5].

It is widely accepted that the quality of the surgical margin is of prime importance for local control [6–8]. To compare results from different series and to evaluate other treatment modalities for local control, a strict definition of the margin assessment procedure is needed. 

Different routines for margin assessment are described [5, 8–12]. Most studies report a margin assessed by the surgeon and validated by the pathologist or jointly assessed by the two. In recent years, it has also been more common to report whether the pathologist has found microscopic tumor tissue at the specimen perimeter, defining a positive or negative margin [13].

Not all studies detail these procedures. The surgeon may measure the thickness of the smallest margin of surrounding tissue on the fresh specimen, omitting areas of smaller distances where there is fascial coverage. The pathologist may measure the thickness of the cuff macroscopically on fresh or formalin-fixed specimen using a variable amount of slices. Finally, the smallest distance without fascial cover can be measured microscopically as the distance from tumor tissue to an inked surface. The number of slides necessary for the pathologists' conclusion may or may not be stated, even when detailed margin analyses are done [14].

In Scandinavia, a wide surgical margin without radiotherapy was formerly considered adequate for treatment of localized STS. Radiotherapy was applied after surgery with intralesional or marginal margins. From 1998, adjuvant radiotherapy was recommended by the SSG for all deep extramuscular, high-grade sarcomas regardless of surgical margin or size. In the context of evaluating the efficacy of this change of policy regarding local recurrence rate [15], the present study evaluates the validity and reliability of the original assessment of the surgical margin as recorded in the Central Register of the SSG. Furthermore, we wanted to investigate if classification of surgical margin at different institutions adhered to SSG guidelines.

## 2. Materials and Methods

Nine university hospitals with a sarcoma unit report to the Central Register. Compiled between 1986 and 2011, this Register contains data on 8322 bone and soft tissue sarcoma patients. They all had their final treatment for primary tumor at a sarcoma centre in Scandinavia. Detailed guidelines for reporting the variables exist, and these are discussed among local unit coordinators on a yearly basis. During the first five-year period after 1998, 470 patients were treated for a primary, previously not operated, soft tissue sarcoma of the extremity or trunk wall (liposarcoma grade I excluded) at four large Scandinavian institutions. These institutions have contributed 68% of the total content of the Register.

Of the 470 patients, 117 (25%) were randomly selected for blinded reassessment of margins by four sarcoma surgeons representing the above-mentioned institutions. 

The evaluation was based on reports from the operation and the pathology evaluation. The original margin classification as reported to the SSG Central Register was in most cases based on collaboration between the local surgeon and the pathologist.

Guidelines for margin assessment in this study were those sited in “Centralized Registration of Sarcoma Patients in Scandinavia SSG VII: 2 Modified 31 March 1995.”

**Definitions**

The definitions of surgical margins are basically those of the Surgical Staging System (SSS, Enneking et al. 1980) and those given by SSG 1981 in “Adjuvant chemotherapy in soft tissue sarcoma” (Bertil Stener).

 Intralesional: intracapsular, subtotal, piecemeal removal.
 Marginal: the dissection is close to the tumor in one or more places, perhaps all around (shelling out, excisional biopsy). If during an operation, that is intended to be a wide excision, the tumor is unexpectedly exposed in one single place, or if histological examination reveals that the margin is marginal in one single place, the excision should be described as marginal, irrespective of how much healthy tissue is included elsewhere.
 Wide (without fascial containment of the surgical specimen): the tumor is removed en bloc completely surrounded by a safety margin of healthy tissue. The tumor is not completely surrounded by an unbroken fibrous boundary. A wide margin for a subcutaneous sarcoma requires inclusion of the deep fascia beneath the tumor. For a tumor located between muscles, the muscles around the tumor are included in the surgical specimen.
 Myectomy (wide with fascial containment of the surgical specimen): the muscle in which the tumor is located is removed unopened along with its fibrous boundary (sometimes bone).
 Compartmental: the tumor-involved compartment (defined according to SSS) is removed en bloc.

In the SSG, the margin should be assessed on the pathological specimen after fixation in formalin and ink-dying of the surface. The specimen is sliced at maximum 1 cm increments and gross-sections or partial gross-sections are made from areas of closest margin at the surgeon's guidance. The pathology report must also include an evaluation of the quality of the margin and the tumors growth pattern at the periphery.

The SSG originally defined “the cuff of healthy tissue” at around 5 cm. As in other parts of the world there has been a gradual change to acceptance of a smaller cuff. Today, the SSG defines the wide margin as a cuff of at least 10 mm nonfascial tissue surrounding the tumour. 

The panel was blinded for the original assessment when independently scrutinizing the surgical and pathology reports. The surgeons did not evaluate margins from their own institution.

During a second round of assessments, all four surgeons convened, and the 25 cases where they disagreed with each other during the independent round were discussed before a final agreement was reached. The originally recorded margin was then disclosed.

## 3. Results

Among the 117 patients, originally recorded margins were 8 intralesional, 43 marginal, and 66 wide.

**First Round**

In 71% of cases, all three reviewers agreed and in 8% all three reviewers disagreed with the original classification. In 19/117 (16%), one reviewer disagreed, and in 6/117 (5%), two reviewers disagreed. When all reviewers disagreed, disagreement was most frequent when addressing the distinction between marginal and wide margins. Reclassification from marginal to wide and from wide to marginal was equally favored.

**Second Round**

In 17 of 25 cases of disagreement among the surgeons, one reviewer changed opinion after listening to the arguments of the other three. In 5/25 cases, two reviewers changed opinion, and in 3/25, there was no change of a differing opinion.

The opinion changes were not necessarily in favor of the original classification.

**Figure 1:**
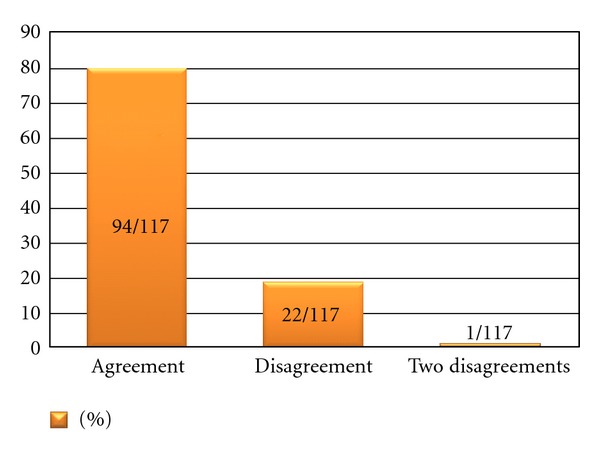
Final agreement among three reviewers with original margin classification. 117 randomly selected cases from four large Scandinavian sarcoma centers, treatment 1998–2003.

**Figure 2:**
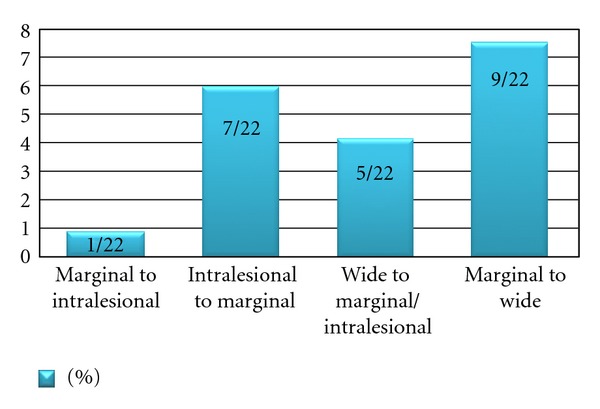
A fraction of patients where all reviewers agreed on a different margin than originally reported to the SSG Register relative to the total amount of cases reviewed (*n* = 117).

The final result was agreement with the original assessment in 80% of cases (Figure 1). After discussing the cases, more reviewers favored a change from marginal to wide margin than vice versa (Figure 2).

After a median followup of 7, 8 years, 11 patients experienced a local recurrence of which the distribution among assessment groups is given in Table 1.

## 4. Discussion

We present two different ways of margin reviewing. An independent approach and a discussion among reviewers. It is not obvious which is the most reliable, and we think both methods convey valuable information.

Margin assessment years after the original recording is not the same as real-time evaluation, even if guidelines are the same. The judgment by the operating surgeon concerning the completeness of resection has elements of quality beyond his written report. This may be illustrated by studies of breast cancer surgeons' ability to determine the margin based on examination of the gross specimen [16–18].

When many hospitals are classifying margins, heterogeneity might be more pronounced than for single institution materials. Among hospitals participating in this study, one institution systematically emphasized the pathologist's measurements of distances on formalin-fixed specimens to differentiate between marginal and wide margins. Consequently, this institution reported 15% less cases of wide margin than the others. Most cases where the panel suggested a change from marginal to wide margin represented patients from this institution. If the reassessment had been carried out without this institution, the panel would have disagreed with the original margin classification in only 7% of cases. This illustrates the need for a large sample size when multicenter margins are evaluated.

The need for carefully defining both study population and how margins are classified is illustrated by local recurrence rates following various margins. Enneking et al. [19] originally reported a 50% local recurrence rate after a marginal margin, 25% after a wide, and 4% after radical resection. Modern series typically have 90% local control after wide margins or after marginal margins with additional radiotherapy [15]. 

None of the intralesional margins in the present study represented cases with gross tumor left. They were all primarily treated at a sarcoma center, and an area of positive margin was typically present only in a small part of the specimen. The distinction between intralesional and marginal margins may, therefore, be of limited prognostic value in this material. From this point of view and without the institution emphasizing the pathologists measurements, the fraction of disagreeing margin assessments of prognostic significance could be regarded to be less than 4%.

Among the cases that eventually had a local recurrence, none had all reviewers agreeing on a different margin than the original one.

Studies of interobserver variability in assessment of surgical margin, attempting a differentiation between marginal and wide margins, are not published. When assessing only the likelihood of residual tumor at the resection margin, two reviewers disagreed on only 1/62 patients [20]. When addressing only positive or negative margins after radical prostatectomy, a very low interobserver variability among 10 pathologists was reported [21]. 

In a resent SSG series of treatment results in STS, the surgical margins were wide or better in 76% of subcutaneous lesions and in 58% of deep-seated lesions [15]. Amputation rate was 7% and had been declining. The rate of wide margins obtained was lower than reported in other series [9, 22, 23]. The local recurrence risk was, however, similar. This might imply that the Scandinavian definition of margins may be somewhat more rigorous.

Since the first SSG protocol for STS adjuvant treatment in 1981, the Surgical Staging System (SSS) has been in use when recording margins in Scandinavia [1]. These principles were also adopted when the SSG Central Register was established in 1987. The SSS recommends the “cuff of healthy tissue,” required for a wide margin, to be as extensive as “is practically possible.” No fixed measurements were originally given, but 3 cm of healthy tissue was required around the biopsy tract, and 5 cm surrounding the tumor on fresh, unfixed specimen was often advocated later. The concept of Enneking's reactive zone omits the need for any measurement. The problem is that the reactive zone is poorly defined, often not visible on histological slides, and many STSs do not have a reactive zone.

In 2006, positive/negative margins were introduced for the SSG Central Register. The intralesional margin is categorized into two types: macroscopic tumor tissue left behind or not. A marginal margin is recorded when the plane of excision passes outside the tumor, but in any part too close to the tumor to merit a wide margin. In other terminologies, the intralesional margin (both types) corresponds to a positive margin whereas the marginal margin corresponds to a negative margin. 

A wide margin is recorded when the excised tumor is surrounded all around by a cuff of healthy tissue or uninvolved fascia. The margin obtained by myectomy is regarded as a subtype of the wide margin and has been applied for strictly intramuscular lesions (not subjected to open biopsy) when the involved muscle, from origin to insertion, is completely removed. 

The necessary thickness of the cuff to merit a wide margin has been discussed during the years. In the latest soft tissue sarcoma protocol (SSG XX) active from 2007, a cuff thicker than 10 mm in a formalin-fixed specimen is considered adequate for a wide margin to be recorded. Future studies may utilize improved methodology when evaluating the adequacy of planning treatment and reporting procedures, for the resection of soft tissue sarcoma [24]. 

## 5. Conclusion

For multicenter studies of prognostic significance of surgical margins, written guidelines for margin assessment are important. The procedure of margin assessment in Scandinavia can be considered valid for measuring the real distance between the tumor surface and the resection margin. It is also reasonably reliable as there is a high level of agreement among sarcoma surgeons evaluating the procedure retrospectively. Considered the element of judgment inherent in all margin assessment, we find this validity and reliability acceptable for using the Scandinavian Sarcoma Group Register for studies of local control.

## Figures and Tables

**Table 1:** 11 Local recurrences and margin assessments.

Number of recurrences relative to type of margin	Original margin assessment	Radiation	Reviewer assessments
4/66	wide	1/4	All reviewers agreed on wide
2/43	marginal	0	All reviewers agreed on marginal
2	marginal	0	Two reviewers intralesional, one marginal
1/8	intralesional	1/1	All reviewers agreed on intralesional
1	marginal	1/1	Two reviewers wide, one marginal
1	wide	0	Two reviewers wide, one marginal
